# Home was not a safe haven: women’s experiences of intimate partner violence during the COVID-19 lockdown in Nigeria

**DOI:** 10.1186/s12905-021-01177-9

**Published:** 2021-01-20

**Authors:** Olufunmilayo I. Fawole, Omowumi O. Okedare, Elizabeth Reed

**Affiliations:** 1 Department of Epidemiology and Medical Statistics, Faculty of Public Health, College of Medicine, University of Ibadan, Ibadan, Nigeria; 2 Division of Health Promotion and Behavioral Science, School of Public Health, San Diego State University, San Diego, CA USA; 3 Center on Gender Equity and Health, Division of Infectious Disease and Global Public Health, University of California San Diego, 9500 Gilman Dr., La Jolla, CA 92093 USA

**Keywords:** COVID-19 pandemic, COVID-19 lock down, Intimate partner violence, Gender based violence

## Abstract

**Background:**

Emergency situations, including epidemics, increase incidence of violence against women, especially intimate partner violence (IPV). This paper describes specific scenarios of IPV reported by women during the COVID-19 pandemic in Nigeria to provide insight for policy and programmatic efforts.

**Methods:**

This paper draws on seven de-identified case reports from organisations serving women experiencing IPV as well as media coverage of IPV cases in Nigeria, between April and May, 2020.

**Results:**

In most cases, reports identified IPV that was occurring prior to the lockdown, but increased in severity or involved new types of violence during the lockdown. The case scenarios included descriptions of many forms of IPV commonly reported, including physical, economic, psychological and sexual violence, often concurrently. Several women also reported threats of being thrown out of their homes by perpetrators, which threatens women’s ability to protect themselves from exposure to COVID-19, but could also leave women stranded with no access to transportation, social services, or other resources during the lockdown. Several women also reported IPV that involved custody of children, as well as IPV that disrupted women’s income generation. IPV was also reported in relation to economic stressors associated with the lockdown. Reports highlight how the lockdown disrupted women’s social support, hindering accessibility of formal and informal sources of help.

**Conclusion:**

The lockdowns in Nigeria may have inadvertently placed women already experiencing partner violence at risk for experiencing more severe violence, new challenges to cope with violent experiences, and other forms of violence, including violence that used the lockdown as a way to threaten women’s security and ability to protect themselves from the virus. Hence, there is need for innovative approaches to support victims, with emphasis on ways in which perpetrators of IPV may be using the threat of COVID-19 to further gain power and control over partners.

## Background

Intimate partner violence (IPV) is a global health problem with one in three women of reproductive age having experienced IPV [1, 2]. IPV is widely accepted in many low- and middle-income countries, including Nigeria, because of traditional norms that support male dominance [3]. In Nigeria, the past-year prevalence of physical, sexual or emotional violence by a partner had been reported in rates as high as 69% [4]. Notably, violence against women in the home may also involve abuse of children as well as other household members [1, 5]. IPV is also associated with a multitude of poor mental, sexual and reproductive health outcomes [6].

Emergency situations, including epidemics, increase incidence of violence against women, especially IPV. The COVID-19 pandemic has been associated with an increase in IPV due to the ways in which control measures were implemented. Studies have documented an upsurge in IPV around the world during the COVID-19 pandemic lockdown (state of isolation or restricted access instituted as a security measure) [7–9]. For instance, the lockdown in the China province of Hubei was associated with more than a threefold increase in cases of IPV [10]. Another report documented 33%, 30% and 25% rise in IPV cases in Singapore, France and Cyprus, and Argentina respectively [9]. Reports from other countries including Brazil, Canada, Germany, Italy, Spain, UK, and the US also substantiate a soar in IPV occurrence and demand for shelter during the COVID-19 lockdown [9, 10].

The situation of heightened IPV related to the lockdown is also apparent in Nigeria. In Nigeria, to control the spread of the virus, the Federal Government declared a total lockdown in two states (Lagos and Ogun) and the Federal Capital Territory, which lasted from the 30th of March, 2020 until the 2nd of May [11]. While the extent of the lockdown varied by state, all states imposed some level of lockdown ordering people to stay at home [12]. During this time of the lockdown in Nigeria, partner violence has been shown to have increased significantly, up to 56%. In the first two weeks of lockdown, IPV cases rose from 346 (March) to 794 (early April) [13]. The Lagos State Domestic and Sexual Violence Response Team reported that hotline calls more than doubled during the lockdown in the state. The response team added extra handlers on its hotlines to cope with the number of calls they are receiving [13].

During COVID-19 lockdowns, existing but limited evidence suggests that the increase in incidence of violence against women, especially IPV, may be because victims remain confined to their homes with perpetrators [8, 9]. The lockdown also meant limited options for women to seek immediate assistance or help (beyond hotline calls), given women’s restricted mobility as well as the limited health, legal, and social service infrastructure available [8, 14]. In addition, COVID-19 lockdown may have decreased informal mechanisms of social support through family and close friends, where in Nigeria (and across the globe), most women are more likely to seek these types of informal support for help with IPV [4, 13, 15–17]. One other major factor attributed to the increase in IPV during the COVID-19 pandemic has included economic stress due to disruption of income and earning power, resulting in reduced access to basic necessities and services [8, 14]. Previous research has documented economic stressors experienced by males to be associated with increased IPV perpetration [18].

Understanding the specific scenarios of IPV women have experienced during the COVID-19 lockdown, including the types and severity of IPV, will be important to better support women experiencing IPV during the COVID-19 lockdown, as well as other similar events in the future. This paper aims to describe women’s experiences of IPV during the COVID-19 pandemic, with a focus on case reports in Nigeria given the high rates of IPV reported in the country.

## Methodology

This paper draws on information retrieved from media coverage and reports from an organisation that respond to violence against women during the lockdown. Seven cases identified from: (1) an organisation serving abused women (n = 4), (2) media reports from the internet (n = 3) are reported. The seven cases reported came from three southern states of Nigeria. The organisation providing data on cases was one of the main one serving women in Southern Nigeria during the lockdown and had worked with victim-survivors prior to the lockdown.

During the lockdown, the organisation shared COVID-19 palliatives (food items) to women in indigenous communities. The organisation also provided contact information for women in need of help to get in touch. Hence, women who experienced abuse during the lockdown were able to obtain support and linked with other sources of help. As part of their service delivery, the organisation usually obtains verbal consent for information provided by clients to be used for research purposes. Thus, informed consent is obtained by the head of the organisation and documented in client’s record. The organization asked clients to consent to their de-identified data or information being used for research purposes. Data was accessed by asking the head of the organisation to discuss specific case summaries seen during the COVID-19 lockdown. Media reported cases, including ‘Twitter’ were identified using Google Search in May 2020. The searches were conducted using the different keywords such as: wife beating, domestic violence, intimate partner violence, physical violence, sexual violence, lockdown, COVID-19, coronavirus, Nigeria. The searches using different keywords returned similar results. Altogether in the electronic media, 7 distinct cases of domestic violence were identified. Only media report of IPV were considered for inclusion and analysis in the present study. Cases (n = 2) were excluded if it was uncertain that they occurred during the lockdown and if they were other forms of interpersonal violence such as; fighting between neighbours and non-partner rape (n = 2). All the cases narrated occurred in States within Nigeria.

All cases were de-identified, no names were given and we changed details of employment, age, family information, and other related information of the case reports. The women who sought help from the NGO organisation were followed up by telephone calls. They were also encouraged to keep in touch to foster access to justice and promote safety. However, it could not be ascertained if all the cases reported from the media were followed up. Ethical approval was waived by the University of Ibadan/University College Hospital (UI/UCH) research ethics committee. This study is a secondary data analysis and involved no direct contact with human participants. It is a review of media reports and records of an organisation serving abused women. Any possible linkage with the clients or organisation were deliberately removed.

A case of IPV during lockdown was defined as any IPV that occurred between March 30 and May 2, 2020, which was the period of total lockdown in most states of the country. Experience of any form of IPV was documented which included:- physical (beating, hitting, choking), sexual (unwanted sexual intercourse, pressure to have sex without the partner’s consent), psychological (abuse, humiliation, threat) or economic (refusing to give financial support for the family upkeep, forcefully taking wife’s money/economic resources).

Data were analysed using qualitative content analysis. The following data were extracted from the reports obtained: (1) Type(s) of IPV experienced during the lockdown (2) Trigger(s) of the IPV (3) How lockdown exacerbated IPV experience (4) Health consequence(s) of IPV (5) Coping strategies with IPV experience during the lockdown.

## Results

Seven cases of IPV were identified through IPV service organizations and media searches. The case scenarios included descriptions of many forms of IPV commonly reported, including physical, economic, psychological and sexual violence, often concurrently. There were also reports of threats to evict women from their home, and in two cases, women were locked out of their houses in the middle of the night. Notably, losing housing security during a pandemic lockdown threatens women’s ability to protect themselves from exposure to COVID-19. It also has the potential to leave women with no access to transportation, social services, or other resources during the lockdown. Several women also reported IPV that involved custody of children, as well as IPV that disrupted women’s income generation. IPV was also reported in relation to economic stressors associated with the lockdown. Reports highlight how the lockdown disrupted women’s social support, hindering accessibility of formal and informal sources of help. (See Table 1 for a summary of each case of IPV identified).

**Table 1:** Case reports of IPV during the COVID-19 lockdown

**Case 1** Mrs A is in her thirties and works in a hospital. She and Mr A had experienced disagreements before the lockdown, but it never involved physical violence, their disagreements were usually settled by family members or religious leaders Two weeks into the lockdown, the disagreements became more serious and involved physical violence. Mr A also threatened to take the children away from Mrs A. At a point, Mrs A was banished from their bedroom to the living room, a place she stayed for few days When the threats to take the children away became intense, Mrs A moved her children to her mother’s place. The movement of the children aggravated her experience of physical violence. Mrs A was forced to rent a small place to save her life and consequently take her children back, but the landlord did not allow her to move in after learning that she is a health worker and feared contracting the COVID-19. Mrs A had no choice but to continue to endure the abuse
**Case 2** Miss B was restricted by the “stay at home” order, which required her to remain in her boyfriend’s house. One morning, the couple had an argument over food shortage in the house, and the boyfriend’s drinking habit. After the argument, Miss B’s boyfriend locked her up in the house and went out till evening. Miss B’s boyfriend returned home drunk, and physically attacked Miss B. The physical violence resulted in a cut in her lips, black eyes, bruises and swelling all over her body. Her aunt had to take her to the health facility for treatment With the support of her aunt, Miss B reported the incident to the police, but they were told to go home and resolve her differences with her boyfriend, especially since the courts were closed and so there is no way to pursue the case legally. Miss B’s aunt was not happy that the boyfriend was let off so lightly and so linked Miss B with an NGO which reported the case again to the Police
**Case 3** Mrs C got married in 2019 and is currently pregnant. Before the lockdown, their young marriage was filled with happiness and love, they both had busy work schedules. The two saw the announcement of the lockdown as an opportunity to have the honeymoon they could not have because of the high demand of her husband’s work. However, Mrs C started to experience physical abuse when she would not accede to her husband’s excessive sexual demands. During one of such events, Mrs C’s head was hit on the floor and then on the wall. Her cries attracted neighbours attention, who then came out and saw her trying to jump to safety from the balcony of their one storey apartment. Mrs C stated that she wanted to go back to her ‘father’s house’. Mrs C also reported that her husband has stopped her from calling her parents or siblings. On one of the following nights, Mr C locked her out (till midnight) of the house for phoning her mother. He had warned her never to call her mother again if she “liked herself”
**Case 4:** This was a sensational story reported on the television and all social media platforms in the country. After a few weeks of the lockdown, Mrs D was seen in a video clip running from her house onto the street dressed with just a cloth wrapped around her body. She was followed by Mr D, who was wearing a pair of shorts and singlet and was trying to coax her back inside their house. Mrs D was seen complaining out loud that she did not want to have sex again. She said that she was engaged in sex with her husband throughout the night, yet her husband insisted on her having sex again. She asked him if he wanted to snuff the life out of her. The neighbourhood was nearly deserted as everyone was lockdown in their homes and it was early in the morning. The few people on the street just looked on
**Case 5** Late in the month of April, the story of Mrs E, a woman in her forties, was reported in the media. Mrs E has four children. She stated that the IPV began after she asked Mr E for money to buy foodstuff to stock up for the lockdown. Mr E refused to give her any money claiming he had bills to settle. Mrs E persisted on her request for money to feed the family. Mr E left the house, on his return he came back with a cutlass and threatened to kill Mrs E He beat Mrs E up so badly and tried to strangle her, but she escaped miraculously. Her friend informed a NGO about her situation who reported the incident to the Police. The Police arrested the Mr E and investigations are on-going
**Case 6** Mrs F has been married for about 5 years and is a mother of four children. Mrs F’s experiences of IPV became aggravated by the lockdown because she could not leave the house and thereby avoid Mr F like she used to do before the lockdown. One day in the last week of April, Mr F threw Mrs F out of the house in the middle of the night but Mrs F refused to leave because there was a curfew in the state and no movement was allowed after 7 pm. Her failure to leave the house resulted in physical abuse by Mr F. Thus, she sustained injuries to her face and arms. Fortunately, the neighbour’s intervened and stopped the beating thereby reducing the injuries she sustained. In the morning, Mrs F moved to her aunt’s house for safety, but the children remained with Mr F. The following day, Mr F locked up Mrs F’s shop that he opened for her. He told the neighbours she was not to manage the shop anymore and asked them to inform him if Mrs F was seen in the shop. Mr F was notified a few days later when Mrs F tried to open the shop for business. Mr F beat Mrs F up again and broke her leg. Mrs F had to be rushed to the hospital. Mr F also demanded that she should come and take ‘her’ children away * It is common for perpetrators of IPV to use children as part of the ways in which they inflict abuse and attempt to make female partners feel degraded, in fear, or otherwise powerless
**Case 7** Mrs G is a school teacher and mother who has been married for many years. In addition to teaching, Mrs G runs a small business to augment her teaching income. As a result of her hard work, she was the main contributor to the family income. Mrs G's husband also works in the education sector. The closure of schools and the business as a result of the lockdown reduced family income and thereby created a financial stress and food insufficiency in the house. Mrs G requested for financial help from a friend outside the country who sent her N30,000 (50 lb) by electronic money transfer. Mrs G asked her husband to collect the money from the bank on her behalf because she did not have an identification card needed to receive the transfer. After Mr G collected it, Mrs G requested for the money from her husband but he refused to give her claiming there was groceries at home and that he needed the money. The husband resorted to verbal abuse in front of their children and neighbours when Mrs G insisted on collecting the money. So she decided to leave the house with her children and stayed with a relative for a few days for safety

We have changed details of employment, names, age, family information, and other related information to keep the identity of individuals confidential and private

## Discussion

This paper highlights the experiences of seven women experiencing IPV during the COVID-19 lockdown. While data indicate that in many countries there has been an increase in IPV associated with the COVID-19 lockdowns [7, 16, 19–21], less is known about the scenarios of IPV occurring and how women’s experiences of IPV may be intersecting with COVID-19 lockdowns or other restrictions. Our findings suggest that the lockdowns in Nigeria may have inadvertently placed women already experiencing partner violence at risk for experiencing more severe violence, new challenges to cope with violent experiences, and other forms of violence, including violence that used the lockdown as a way to threaten women’s security and ability to protect themselves from COVID-19 exposure. Women reported that social isolation and lack of access to formal services inhibited their ability to get support as a result of IPV during the lockdown. Additionally, economic stressors associated with the COVID-19 lockdown were noted by women as aggravating or resulting in IPV. While more research will be needed, these case summaries provide some initial insight to women’s experiences of IPV unique to the COVID-19 lockdown.

The case studies we reported suggest that perpetrators of IPV may be engaging in violence, or threats of violence that could increase women’s risk for COVID-19 exposure, or use the COVID-19 lockdown as a platform to provoke fear and threaten women’s overall security during the pandemic. Cases involved perpetrators threatening to kick victims out of the house and in two cases, the perpetrator locked the woman out of her house during the stay at home order. Such threats of homelessness may be especially fear provoking during the pandemic lockdown, where everything was closed and there was no opportunity to obtain transportation or to reach formal or informal support sources. Future research with larger samples, including quantitative surveys, will be needed to better understand whether these types of threats were common among women experiencing IPV during the COVID-19 lockdown. While we identified threats to women’s housing security, there may likely be other types of scenarios where perpetrators used the COVID-19 pandemic or lockdown to provoke fear and threaten women’s security. This is an important area for future research that is new to the literature on IPV, and has not been identified in previous work.

Apart from the social stress of the pandemic, our findings suggest that economic stressors may underlie increased reports of IPV perpetration during the COVID-19 pandemic due to disruption in income and earning power [22]. Our findings indicated that money worries and food insufficiency created tension that resulted in conflict and IPV. The association between economic stressors and IPV have been demonstrated in previous work, and thus, are well-aligned with our findings [18, 23, 24]. One study conducted among couples in India found that debt was associated with increased IPV perpetration by male partners [18].

Our findings suggest that the lockdown isolated women and reduced opportunities for them to disclose the abuse or to receive necessary support services or other resources. Notably, work sites and religious organizations which often offer critical emotional support and provide opportunity for a “reprieve” for victims were no longer available at this time leaving women nowhere to go for support [25]. A few victims went to the health facility to obtain care but were not able to receive all the support they needed. The early identification and timely management of cases may halt progression of abuse, provide opportunity for the development of safety plans and thereby reduce the consequences of violence [14]. In addition, more work may be needed to reinforce informal support for IPV, including neighbours or other community member intervention to stop IPV when it occurs in public. In Nigeria, IPV is accepted as a family issue that requires no outside interference [26]. Generally, the community only intervenes when IPV is life-threatening or there is likelihood of having legal implications as demonstrated in two cases.

Many of the cases were reported by the Non-Governmental Organisation (NGO). NGOs provide guidance to victims and representatives who can link victims with the legal system [13]. Government agencies and ministries do not often support victims, while the Police often dismiss IPV incidents as a family mater which should be settled by the elders [26, 27]. In the absence of courts to provide restraining orders or family/friends victims can go to for safety, NGOs are very important because of their shelters and extensive supports to victims of violence. However, at the present time they are experiencing challenges in respect of staffing and budgetary constraints—all of which might further affect care to victim-survivors [28].

Victims of IPV in Nigeria and many low income countries are disadvantaged because of non-availability of shelters that can provide a safe space. In many states of Nigeria, there are no public shelters for victims of abuse, and where they exist, they are grossly underfunded [13]. NGOs and a few women societies provide the few shelter services available to women. However, there is constraint to their activities during the lockdown, because they are not included as essential service providers [7, 8]. Governments therefore need to make provision for shelters and referrals centers a priority to help victims of abuse, especially during emergencies when health care providers may be overburdened and unavailable.

The failure of police officers to address cases of IPV is a cause for concern. The courts were closed because of the lockdown; therefore, the police did not wish to prosecute cases. Enforcing the laws to reduce the spread of the virus resulted in deployment of many law enforcement officers into the community, and as such were not readily available to take report of abuse [13]. Similarly, the need to decongest prisons during the lockdown may make the police not to arrest perpetrators of IPV [8]. The law courts need to be able to provide services during this and similar emergencies. When victims are confident that perpetrators will be prosecuted and there is no fear of reprisal attack, there will be improved reporting.

IPV requires a careful combination of legal measures (e.g. arrest of perpetrators, prosecution by judiciary, safety orders), societal responses (e.g. community responses, advocacy, shelters) and heightened awareness by frontline care providers such as medical and social services, where victims can present with physical or psychological trauma, sexual and reproductive health complications, neglect or other squeal of abuse, including mental health services [25]. The use of the telephone or online platforms has been recommended as an effective and discrete method for victims to reach help on time at a time when face-to-face interaction is limited [29, 30], but even this may be a challenge for some victims, hence there is a need to improve public awareness and response. Our findings suggest that all of these services need to be heightened during emergencies associated with increases in IPV, such as the COVID-19 lockdown. However, more work is needed to further understand how to better serve victims of IPV during these times of community emergency.

Our case report has some limitations; the reported cases were small in number and mostly from the urban areas and thus, may not reflect the experiences of women in the rural areas. Generally, the rural areas have less access to public services such as police, law enforcement agencies and shelters The prevalence of IPV against women is usually higher in rural compared to the urban areas [31] and this may worsen during the pandemic. Also, the report of abuse from the media platform may be sensationalized. Despite this, these case reports provide initial insight into the types of scenarios experienced by women reporting IPV during the lockdown. Even with a small number of cases and less detailed information than would otherwise be obtained via other research methods (e.g. in-depth interviews), we still found common themes across these 7 cases, including new forms of violence that may be specific to the COVID-19 lockdowns. Future research studies will be needed that employ surveys or qualitative in-depth interviews and that recruit larger, more representative samples of women across various geographic locations in Nigeria to expand upon and confirm these findings.

## Conclusion

Even with the ease of the lock down, the social and economic impact of the pandemic are likely to continue for an extended period of time, hence the stress and risk factors for IPV are likely to continue. It is imperative for government and NGOs to support women during periods of emergencies to ensure they have access to health care services and judicial support. Also, family and community members need to be more involved and supportive of abused women during period of emergencies. There is need for more research and innovative approaches to support victims of IPV.

## Data Availability

The datasets used and/or analysed during the current study are available from the corresponding author on reasonable request.

## Abbreviations

COVID-19: Corona virus of 2019
IPV: Intimate Partner Violence
NGO: Non-governmental Organisation
UK: United Kingdom
US: United States
WHO: World Health Organisation
